# The P2X_7_R is a crucial target for Angiotensin II-induced myocardial ferroptosis and remodeling

**DOI:** 10.1007/s11302-024-10048-5

**Published:** 2024-09-21

**Authors:** Abdel-Aziz S. Shatat

**Affiliations:** Department of Pharmacology and Toxicology, Faculty of Pharmacy, Al-Azhar University, Cairo, Egypt

**Keywords:** P2X7 Receptor, Angiotensin II, Myocardial ferroptosis, Cardiac remodeling

## Abstract

Ongoing cardiac remodeling can lead to negative outcomes, such as cardiac failure and diminished myocardial function, although the remodeling process initially protects the heart as a compensatory mechanism[1] . Importantly, ferroptosis appears to be a critical process in the development of cardiac disease. In a recent publication in *Redox Biology*, (Zhong et al. [2] showed that reactive oxygen species (ROS) generation and cardiac ferroptosis may be the mechanisms underlying angiotensin II (Ang II)-induced cardiac remodeling, as well as that ferroptosis is required for heart impairment and cardiac dysfunction induced by Ang II. Moreover, this study provides evidence that Ang II increases the expression of P2X7 receptors (P2X7R) in cardiac tissues and that both silencing and pharmacological inhibition of P2X7R significantly inhibited Ang II-induced ferroptosis and hypertrophy. Also, this work confirmed that P2X7R deficiency mitigated the Ang II-induced deterioration of cardiac injury in mice fed an iron-rich diet. Most interestingly, this study revealed that Ang II directly interacts with the P2X7R to activate and induce nucleocytoplasmic shuttling of human antigen R (HuR), which in turn controls the stability of the mRNA of heme oxygenase 1 (HO-1) and GPX4 and subsequent ROS production, which translated to induction of myocardial ferroptosis and remodeling.

## Commentary

Fibrosis, myocyte loss and hypertrophy of cardiomyocytes are the usual symptoms of cardiac remodeling. Moreover, adverse events, such as reduced myocardial function and heart failure, can arise from prolonged cardiac remodeling. Importantly, oxidative stress, inflammation, mechanical interference and neurohumoral activation, especially Ang II activation, frequently cause pathological cardiac remodeling [3]. A recently identified type of cell death involving iron and lipid peroxidation is ferroptosis. Most interestingly, A critical form of cell death in cardiomyocytes, ferroptosis plays a pivotal role in the occurrence of doxorubicin-induced cardiotoxicity [4]. P2X7R activation plays a pivotal role in many aspects of cardiovascular diseases, particularly in the development of atherosclerosis, myocardial ischemia injury and myocardial infarction through ROS generation and proinflammatory cytokine release [5]. Understanding the molecular mechanisms underlying cardiac remodeling and identifying novel targets to avert unfavorable cardiac remodeling and heart failure are imperative given the detrimental effects of chronic cardiac hypertrophy.

Zhong et al. first demonstrated that Ang II significantly decreased the expression of GPX4, a biomarker of ferroptosis; in contrast, it increased the expression of HO-1, which catalyses the breakdown of heme to produce biliverdin, ferrous iron and carbon monoxide, and 4-hydroxynonenal (4-HNE), a putative molecular marker of ferroptosis, in cardiac tissues. Also, serum and cardiac tissue iron levels, as well as ROS production (indicated by upregulation of the lipid peroxidation marker, malondialdehyde (MDA), increased superoxide dismutase activity and downregulated glutathione levels) were markedly elevated in response to Ang II injection. Importantly, the previous cardiac changes were associated with marked cardiac dysfunctions. Moreover, the authors then confirmed that a high iron diet (HID) is required to initiate cardiac ferroptosis because it caused cardiac injury, as evidenced by a rise in the heart-to-body weight ratio (HW/BW) and a decrease in ventricular systolic function. These data suggest that Ang II-induced cardiac remodeling may be mediated via cardiac ferroptosis and ROS production [6].

This study then showed that treatment of mice with an inhibitor of ferroptosis, such as deferoxamine (DFO) or ferrostatin-1 (Fer-1), markedly improved systolic dysfunction of the heart caused by Ang II, reduced an elevated serum iron level and corrected the anatomical abnormalities seen in the hearts induced by Ang II. Moreover, Zhong et al. demonstrated that the Ang II-mediated cardiomyocyte enlargement was markedly improved. Furthermore, Ang II-induced prohypertrophic protein elevation (represented by β-MYHC and ANP) and an increase in profibrotic markers (COL-1, TGF-β, and MMP9) in the mice hearts, as well as H9c2 cardiomyocytes and primary rat cardiomyocytes, were dramatically reduced when treated with either Fer-1 or DFO. These data collectively demonstrated that ferroptosis is required for heart impairment and cardiac dysfunction induced by Ang II [7].

RNA-seq data, as well as experimental assays, demonstrated that application of Ang II to mouse heart tissue, primary cardiomyocytes and H9c2 cardiomyocytes, elevated P2X7R mRNA levels and significantly higher P2X7R mRNA and protein levels, respectively. Moreover, this study confirmed that the primary cell type responsible for P2X7R expression in cardiac tissues induced by Ang II is cardiomyocytes. These results collectively imply that P2X7R expression is primarily elevated by Ang II in cardiomyocytes. Furthermore, the functional involvement of P2X7R in Ang II-induced heart dysfunction and heart impairment in mice was investigated by using male P2X7R knockout mice (P2X7R^−^/^−^). Most importantly, the data suggested that Ang II-induced cardiac dysfunction and heart impairment were not seen in mice lacking P2X7R [8].

Additionally, in contrast to the Ang II-P2X7R-/-mice, hearts that were exposed to Ang II showed aberrant structures, such as amorphous myofibers and enlarged myofiber cross-sections. Also, the cardiac tissue levels of ANP, β-MYHC, COL-1, and TGF-β, in addition to the accumulation of collagen in the cardiac interstitium, were markedly decreased in Ang II-P2X7R^−^/^−^ mice as compared to control mice. All of these results point to a significant improvement in cardiac fibrosis and hypertrophy in mice given Ang II when P2X7R were absent [8]. Furthermore, the previous findings were confirmed by pretreatment of cultured primary cardiomyocytes with the selective P2X7R antagonist, A438079, which abolished the elevation in COL-1, MMP9, and TGF-β, in addition to ANP and β-MYHC induced by Ang II [9]. Also, P2X7R silencing significantly attenuated the Ang II-induced upregulation of hypertrophic and profibrotic indicators, as demonstrated by a reduction in COL-1, MMP9, TGF-β, ANP, and β-MYHC expression. These findings provide additional proof that P2X7R silencing and pharmacological inhibition mitigate fibrosis and hypertrophy brought on by Ang II in cultured cardiomyocytes [10].

Moreover, the reduction in GPX4 and rise in cardiac tissue levels of HO-1 and 4-HNE induced by Ang II were eliminated in P2X7R^−^/^−^ mice. Also, Fe^2+^ levels in cardiac tissues were increased following Ang II injection, but this effect was reversed by P2X7R deficiency. Importantly, this suggests that blocking P2X7R can inhibit Ang II-induced cardiac ferroptosis in mice [11]. Additionally, this study demonstrated that the mice treated with Ang II + HID showed significant declines in ejection fraction and fractional shortening and subsequent increases in cardiac fibrosis as compared to control (HID alone-treated mice); however, P2X7R knockout abolished heart systolic dysfunction induced by Ang II + HID, confirming that P2X7R deficiency mitigated the Ang II-induced escalation of cardiac injury in mice fed an iron-rich diet. Furthermore, pre-incubation of H9c2 cells with the selective P2X7R inhibitor, A438079, significantly reversed the alterations in the levels of HO-1 and GPX4 proteins as well as superoxide dismutase activity, glutathione levels, and MDA in Ang II-stimulated cells. Importantly, Zhong et al. showed that after Ang II stimulation, the potential of the mitochondrial membrane in H9c2 cells was decreased, and this effect was then restored by A438079.

Regarding the underlying molecular mechanisms of P2X7R blockade-induced ferroptosis inhibition, this work demonstrated that cardiomyocytes that had been treated with Ang II and P2X7R siRNA, had a shorter half-life of HO-1 mRNA and longer half-life of GPX4 mRNA. Thus, P2X7R silencing enhanced the stability of GPX4 mRNA, while decreasing that of HO-1 mRNA. Subsequently, HuR expression was significantly elevated in the Ang II-treated mice hearts, while in Ang II-treated, P2X7R knockout mice, this expression was restored. Furthermore, Ang II increased the amounts of HuR in H9c2 cells and caused a noticeable rise in the movement of HuR from the nucleus into the cytoplasm (HuR nucleocytoplasmic shuttling), which P2X7R siRNA significantly inhibited. These findings revealed that cytoplasmic HuR expression and accumulation were efficiently controlled by P2X7R deficiency [12]. Also, the authors discovered that in Ang II-treated H9c2 cells, the efficient reduction of HuR via HuR siRNA also enhanced the decline in HO-1 mRNA and decreased mRNA degradation of GPX4. Furthermore, HuR directly interacted with the mRNAs of HO-1 and GPX4. Following the application of P2X7R siRNA, HuR's interaction with GPX4 mRNA increased, while that with HO-1 mRNA decreased. These findings provided credence to the theory that P2X7R blockade modified HuR's binding to the corresponding mRNAs, thereby controlling the mRNA stability of HO-1 and GPX4 [13].

Finally, regarding the molecular connection between Ang II and P2X7R, Zhong et al. found that H9c2 cells were immunofluorescence stained for both the biotinylated form of Ang II (Bio-Ang II) and P2X7R after being treated with either biotin or Bio-Ang II. Colocalization of these proteins on the cell surface suggests that P2X7R and Ang II might have interactions. Additionally, pull-down studies were conducted with primary cardiomyocyte protein lysates and the Ang II-treated mouse heart. The outcomes showed that Bio-Ang II bound to the P2X7R protein in these lysates. Moreover, the extracellular domain of the purified P2X7R protein was directly bound by Ang II (K_D_ = 9.63 nM), according to biolayer interferometry analysis. These results show that Ang II directly interacts with P2X7R to increase the expression of that protein. [14, 15].

To conclude, this work provides evidence that Ang II, through direct activation of P2X7R, can induce the expression and nucleocytoplasmic shuttling of HuR, which subsequently modulates the stability and expression of HO-1 and GPX4 mRNA to promote ROS generation, myocardial ferroptosis, and remodeling.

## Data Availability

No datasets were generated or analysed during the current study.
